# The Human Endolymphatic Sac and Inner Ear Immunity: Macrophage Interaction and Molecular Expression

**DOI:** 10.3389/fimmu.2018.03181

**Published:** 2019-02-01

**Authors:** Charlotta Kämpfe Nordström, Niklas Danckwardt-Lillieström, Göran Laurell, Wei Liu, Helge Rask-Andersen

**Affiliations:** ^1^Section of Otolaryngology, Department of Surgical Sciences, Uppsala University Hospital, Uppsala, Sweden; ^2^Section of Otolaryngology, Department of Surgical Sciences, Head and Neck Surgery, Uppsala University Hospital, Uppsala, Sweden

**Keywords:** human, cochlea, macrophages, IBA1, structured illumination microscopy

## Abstract

**Background:** The endolymphatic sac (ES) is endowed with a multitude of white blood cells that may trap and process antigens that reach the inner ear from nearby infection-prone areas, it thus serves as an immunologic defense organ. The human ES, and unexpectedly the rest of the inner ear, has been recently shown to contain numerous resident macrophages. In this paper, we describe ES macrophages using super-resolution structured fluorescence microscopy (SR-SIM) and speculate on these macrophages' roles in human inner ear defense.

**Material and Methods:** After ethical permission was obtained, human vestibular aqueducts were collected during trans-labyrinthine surgery for acoustic neuroma removal. Tissues were placed in fixative before being decalcified, rapidly frozen, and cryostat sectioned. Antibodies against IBA1, cytokine fractalkine (CX3CL1), toll-like receptor 4 (TLR4), cluster of differentiation (CD)68, CD11b, CD4, CD8, and the major histocompatibility complex type II (MHCII) were used for immunohistochemistry.

**Results:** A large number of IBA1-positive cells with different morphologies were found to reside in the ES; the cells populated surrounding connective tissue and the epithelium. Macrophages interacted with other cells, showed migrant behavior, and expressed immune cell markers, all of which suggest their active role in the innate and adaptive inner ear defense and tolerance.

**Discussion:** High-resolution immunohistochemistry shows that antigens reaching the ear may be trapped and processed by an immune cell machinery located in the ES. Thereby inflammatory activity may be evaded near the vulnerable inner ear sensory structures. We speculate on the immune defensive link between the ES and the rest of the inner ear.

## Introduction

**Figure 1:**
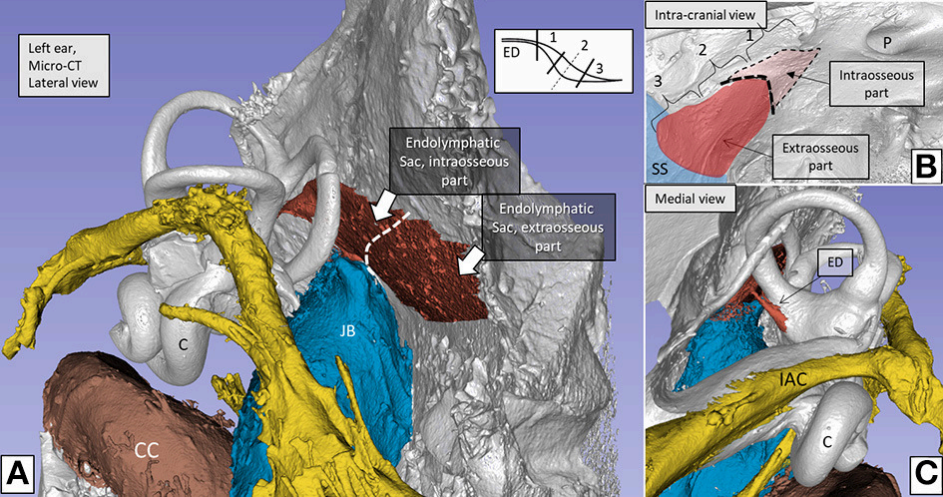
**(A)** Micro-CT and computer 3D reproduction of a left human inner ear silicon cast. The cochlea **(C)** and semicircular canals are seen together with the vestibular aqueduct (red). Facial nerve canal is yellow and veins are blue. The vestibular aqueduct (red) houses the endolymphatic duct (ED) and sac. The sac consists of an intraosseous part located inside the temporal bone and an extra-osseous part located on the posterior slope of the petrous pyramid within the dura. **(B)** The sac exits through an opening in the bone (external aperture of the VA, interrupted line in **A,B**), and it often extends over the sigmoid sinus (SS). **(C)** The ED connects the sac with the rest of the inner ear. The intra- and extra-osseous parts of the sac were investigated in the present study. The sac is divided into a proximal (1), an intermediate (2), and a distal part (3). 1 and 3 have a smooth, single-cell layered epithelium, while the intermediate part (2) has a rugose multi-layered epithelium with secretory-like tubules. Demonstration of the bony surface in **(B)** is possible by using a transparency paradigm within the 3D program. C, Cochlea; JB, Jugular bulb; SS, Sigmoid sinus; IAC, Internal auditory canal; P, Internal acoustic porous; CC, Carotid canal.

The inner ear is segregated by a blood/labyrinth barrier and, like the brain, was generally thought to be immunologically inactive. Recent studies, however, have shown that a large population of IBA1-expressing macrophages reside in the human inner ear (1, 2). The cells have also been found to be present in the endolymphatic sac (ES), a separate portion of the inner ear located away from the cochlea and vestibular organs, which are related to hearing and balance. The ES is a part of the membranous labyrinth and is located in the petrous bone and in a dura duplicate near the cerebellum. The ES is connected to the rest of the inner ear by a filiform endolymphatic duct (ED, diameter 0.1–0.2 mm) that runs to the ES in a bone channel called the vestibular aqueduct (VA). For clarity, the ED and ES is shown in a 3D reconstruction of a human inner ear cast of the Uppsala collection (3) (Figure 1). The ED and ES are generally thought to monitor homeostasis of the endolymph fluid surrounding the sensory hair cells. The fascinating ES has challenged ear researchers for years, and its function remains largely unknown.

Macrophage-lymphocyte interaction and mature plasma cells were earlier described in the guinea pig ES, using transmission electron microscopy (4–6), which supports the notion of local immune responsiveness in the human ES. Arnold et al. (7) found IgA both in the lumen of the human ES and in perisaccular plasma cells and Bui et al. (8) described subpopulations of lymphocytes, macrophages, and plasma cells. The existence of an immunologic route from the middle to the inner ear and from there to the ES was postulated by Ikeda and Morgenstern (9). The ES contains lymphocytes and plasma cells; and it is thought to act as an immunologic defense organ for the inner ear (4, 5, 8, 10). This idea was supported by Danckwardt-Lillieström et al. (11) who found microorganisms (*Mycoplasma pneumoniae*) in the lumen of the human ED, with signs of being processed. Further evidence of potential immune mechanisms was demonstrated by ablation of the ES, which lead to diminished secretion of specific antibodies in the perilymph and reduced immune activity after inner ear antigen challenge (6, 10, 12–16). Tomiyama and Harris (6) suggested that the ES plays a role for both systemic and local antibody responses. Their findings indicated that certain inner ear disorders may be immune-system related and treatable with immunosuppressive agents (10).

Moreover, Möller et al. (17) studied the human ES and found gene expression for both the cellular and humoral innate immune-system, including toll-like receptors (TLR) 4 and 7, beta-defensin, and lactoferrin. These findings provided molecular evidence of an immunological capacity of the ES to recognize and process antigens for immune responses. Beta-defensin and lactoferrin are potent molecules against invading pathogens and were found to be expressed by the ES epithelium, which strongly supported the authors' view that the ES acts as an immunological entity of the inner ear.

Here, we used super-resolution structured illumination microscopy (SR-SIM), together with confocal microscopy to analyze the molecular structure of the human ES and its possible role for inner ear immunity. Freshly fixed human ESs and archival cochleae were analyzed after ethical permission was obtained. The tissue was processed with primary antibodies against IBA1, MHCII, CX3CL1 (fractalkine), CD68, CD4, CD8, CD11b, TLR4, vimentin, and type IV collagen. High-resolution fluorescence microscopy showed MHCII expressing macrophages and interaction with neighboring CD68, CD4, and TLR4 positive cells. The possibility of a functional link between the macrophage systems in the ES and inner ear sensory organs (2, 18–20) is discussed.

## Materials and Methods

### Ethics Statement

The study of discarded human tissue was approved by the local ethics committee (Etikprövningsnämnden Uppsala, no. 99398, 22/9 1999, cont, 2003, no. C254/4; no. C45/7 2007, Dnr. 2013/190), and patient consent was obtained. The study adhered to the rules of the Declaration of Helsinki. Archival sections from adult cochleae were used (21, 22).

### The Human Vestibular Aqueduct

Fresh tissue samples of the human ES were collected during surgery for vestibular schwannoma using the trans-labyrinthine approach. No data on age, gender, or audiometric results were retrieved. Five ESs with surrounding bone tissue were dissected using diamond drills of various sizes. A thin shell of bone was saved around the ES in order to protect the epithelial surface. Both the intra- and extra-osseous parts of the ES were analyzed. The ED and the part of the ES located on the sigmoid sinus could not be investigated. The first part of the ES is named “proximal,” the second part, “intermediate,” and the third part, “distal” (1–3 in Figure 1B). The intermediate part is also termed “rugose” since its epithelium is folded and contains secretory-like epithelial tubules (23). In the operating room the tissue was immediately placed in 4% paraformaldehyde in PBS [1.06 mM KH2PO4, 2.97 mM Na2HPO4, 155.17 mM NaCl, pH 7.4 (ThermoFisher, cat no 10010-015)]. After a 24-h period spent in fixative, the specimens were washed in PBS and then placed in 0.5 M Na-ethylene-diamine-tetra-acetic acid (EDTA) solution (Medicago AB, Uppsala, Sweden) buffered in PBS to pH 7.2 for decalcification. The EDTA solution was changed every 4–5 days until the decalcification process was completed, which took ~3 weeks. The decalcified ESs were rinsed with PBS and placed in 20% sucrose solution dissolved in PBS overnight at 4°C. The ESs were embedded in Tissue-Tek (OCT Polysciences) for frozen sections. The ESs were rapidly frozen and sectioned at 8–10 μm using a Leica cryostat microtome. The frozen sections were collected onto glass slides (SuperFrost® Plus, Menzel-Gläzer, Braunschweig, Germany) and stored below −70°C before immunohistochemistry was conducted.

### Antibodies and Immunohistochemistry

Table 1 shows the series of antibodies used in the present study. IBA1 polyclonal antibody (Thermo Fisher Scientific) from rabbit was used at a dilution of 1:100. The specificity of the antibody was proven by IBA1 antibody blotting (24). The fractalkine antibody used was a monoclonal antibody (1:100, mouse, MAB3651, R&D Systems). This antibody specificity was verified by western blotting (25). The CD68 antibody used was a monoclonal antibody (1:50, mouse, NB100-683, Novus), and the specificity was proven by western blotting (26). For the following antibodies, specificity was proven by the following:

- MHCII—flow cytometry (27).
- TLR4—western blot (www.thermofisher.com).
- CD11b—flow cytometry (28).
- CD4—neutralization (29).
- CD8α–flow cytometry (30).

**Table 1:** Antibodies used in the present study.

**Antibody**	**Type**	**Dilution**	**Host**	**Catalog number**	**Producer**
IBA1	Polyclonal	1:100	Rabbit	PA5-27436	Thermo Fisher, Waltham, USA
CD68	Monoclonal	1:50	Mouse	NB100-683	Novus, Littleton, USA
MHCII	Monoclonal	1:100	Mouse	MA5-11966	Thermo Fisher, Waltham, USA
Collagen IV	Polyclonal	1:10	Goat	AB769	Millipore, Burlington, USA
CX3CL1	Monoclonal	1:50	Mouse	MAB3651-100	R&DSystems, Minneapolis, USA
TLR 4	Oligoclonal	1:10	Rabbit	710185	Thermo Fisher, Waltham, USA
Vimentin	Monoclonal	1:50	Mouse	V5255	Sigma-Aldrich, Saint Louis, USA
CD11b	Monoclonal	1:50	Rabbit	AB52478	Abcam, Cambridge, UK
CD4	Polyclonal	1:150	Goat	AF-379-NA	R&DSystems, Minneapolis, USA
CD8α	Monoclonal	1:100	Mouse	MAB1509	R&D systems, Minneapolis, USA
Anti-Mouse IgG (H+L), Alexa Fluor® 555	Polyclonal	1:400	Goat	A21422	Invitrogen, Carlsbad, USA
Anti-Rabbit IgG (H+L), Alexa Fluor® 488	Polyclonal	1:400	Goat	A11008	Invitrogen, Carlsbad, USA
Anti-Goat IgG (H+L), Alexa Fluor® 488	Polyclonal	1:400	Donkey	A21432	Invitrogen, Carlsbad, USA
Anti-Mouse IgG (H+L), Alexa Fluor® 488	Polyclonal	1:400	Donkey	A21202	Invitrogen, Carlsbad, USA
Anti-Rabbit IgG (H+L), Alexa Fluor® 555	Polyclonal	1:400	Donkey	A31572	Invitrogen, Carlsbad, USA
Anti-Goat IgG (H+L), Alexa Fluor® 647	Polyclonal	1:400	Donkey	A-21447	Thermo Fisher, Waltham, USA

Antibody against collagen IV was used to discriminate the basal lamina and antibody against vimentin to visualize the epithelial cells (31). The immunohistochemistry procedures performed on the sections have been described in previous publications (22, 32). Briefly, the slide-mounted sections were incubated with an antibody solution under a humidified atmosphere at 4°C for 20 h. After rinsing with PBS three times for 5 min each, the sections were incubated with secondary antibodies conjugated to Alexa Fluor 488 and 555 (Molecular Probes, Carlsbad, CA, USA) for 2 h at room temperature. The primary and secondary antibodies were diluted in 2% bovine serum albumin (BSA) dissolved in PBS. The sections were then counter-stained with the nuclear stain DAPI (4′, 6-diamidino-2-phenylindole dihydro-chloride) for 5 min, were rinsed with PBS (3 × 5 min), and were mounted with Vectashield (Vector Laboratories, Burlingame, CA, USA) mounting medium. The sections were rinsed with PBS (3 × 5 min) and mounted with ProLong™ Gold Antifade Mountant (Invitrogen, Carlsbad, CA, USA) as well as the specified cover glass (Mänzel-Gläser, Braunschweig, Germany) required for optically matching the microscope objectives.

Primary and secondary antibody controls and labeling controls were used to exclude endogenous labeling or reaction products (33). The control sections were incubated with 2% BSA, omitting the primary antibodies. The control experiment revealed no visible staining in any structure of the cochleae. Both wide-field and confocal fluorescence imaging software exhibited sensitive fluorescent saturation indications, thereby avoiding overexposure. In addition, archival sections of human cochlea were analyzed according to the methods described in Liu et al. (2).

### Imaging and Photography

The stained sections were first investigated with an inverted fluorescence microscope (Nikon TE2000) equipped with a spot digital camera with 3 filters (for emission spectra maxima at 358, 461, and 555 nm). Image-processing software (NIS Element BR-3.2, Nikon), including image merging and a fluorescence intensity analyzer, was installed on a computer system connected to the microscope. For the laser confocal microscopy, we used the same microscope equipped with a three-channel laser emission system. The optical scanning and image-processing tasks were performed using the Nikon EZ-C1 (ver. 3.80) software and included the reconstruction of the Z-stack images into projections and 3-D images. The SR-SIM was performed using a Zeiss Elyra S.1 SIM system and a 63x/1.4 oil Plan-Apochromat objective (Zeiss), a sCMOS camera (PCO Edge), and the ZEN 2012 software (Zeiss). Multicolor SR-SIM imaging was achieved with the following laser and filter setup:

- first channel: 405 nm laser excitation and BP 420–480 + LP 750 filter
- second channel: 488 nm laser excitation and BP 495–550 + LP750 filter
- third channel: 561 nm laser excitation and BP 570–620 + LP 750 filter
- forth channel: 647 nm laser excitation and LP 655 filter

To maximize the image quality, five grid rotations, and five phases were used for each image plane and channel. The grid size was automatically adjusted by the ZEN software for each wavelength of excitation. The SR-SIM images were processed with the ZEN software using automatic settings and theoretical point spread function (PSF) calculations.

From the SR-SIM dataset, 3-D reconstruction was performed with Imaris 8.2 (Bitplane, Zürich, Switzerland). A bright-field channel was merged with fluorescence to visualize the cell borders. The microscope is capable of achieving a lateral (X–Y) resolution of ≈100 nm and an axial (Z) resolution of ≈300 nm (34). The resolution of the SIM system in BioVis (Uppsala) was measured with sub-resolution fluorescent beads (40 nm, Zeiss) in the green channel (BP 495–550 + LP750). An average PSF value was obtained from multiple beads with the built-in experimental PSF algorithm of the ZEN software. The typical resolution of the system was 107 nm in the X–Y plane and 394 nm in the Z plane. 3-D reconstructions of collagen IV and IBA1 protein expression were conducted. Both signals were reconstructed by a surface rendering mode using the Imaris 8.2 software.

SIM or structured illumination microscopy is a wide-field technique that is based on the Moire effect of interfering fine striped patterns of excitation with sub-diffraction features in the sample emission. This can be compared with the confocal technique where the fluorescence light is detected only at the focal plane. The resolution is twice as big and offers better possibilities to demonstrate proteins at a subcellular level. Combined with confocal microscopy the techniques allow overviews of protein distribution in the tissue as well as a more detailed cellular localization.

## Results

Maintaining the bony wall of the VA at drilling resulted in improved structural preservation of the vulnerable ES tissue, which is well suited for SR-SIM immunohistochemistry after decalcification. The pattern of expression of all the proteins stained for in the study was similar in all 5 specimens.

### IBA1 and Fractalkine Expression

**Figure 2:**
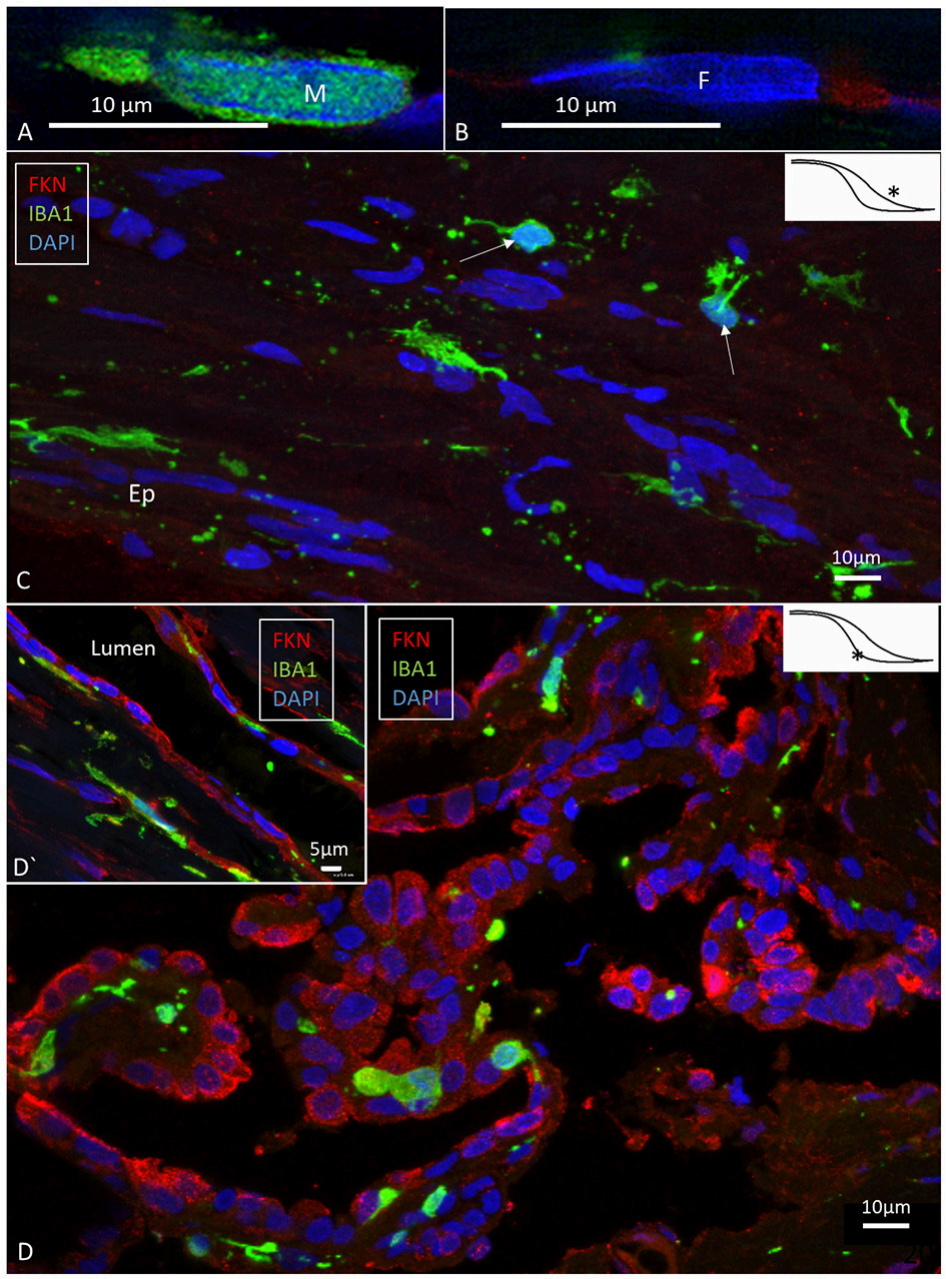
SR-SIM **(A,B)** and confocal fluorescence microscopy **(C,D)** of the human ES. **(A,B)** show the difference in nuclear expression of IBA1 protein in macrophages **(A)** and fibrocytes **(B)** in the peri-saccular tissue. **(C)** The sub-epithelial tissue in the intraosseous part of the ES displays a large number of variously shaped IBA1-positive cells. In two of these cells (arrows), the typical nuclear staining can be seen. Several thin cross-sectioned ramifications of IBA1 cells are visible. **(D)** The epithelium also contains a large number of IBA1 cells, located both between and beneath epithelial cells (inset **D′**). The epithelial cells express the chemokine fractalkine. ^*^Shows location in the ES.

**Figure 3:**
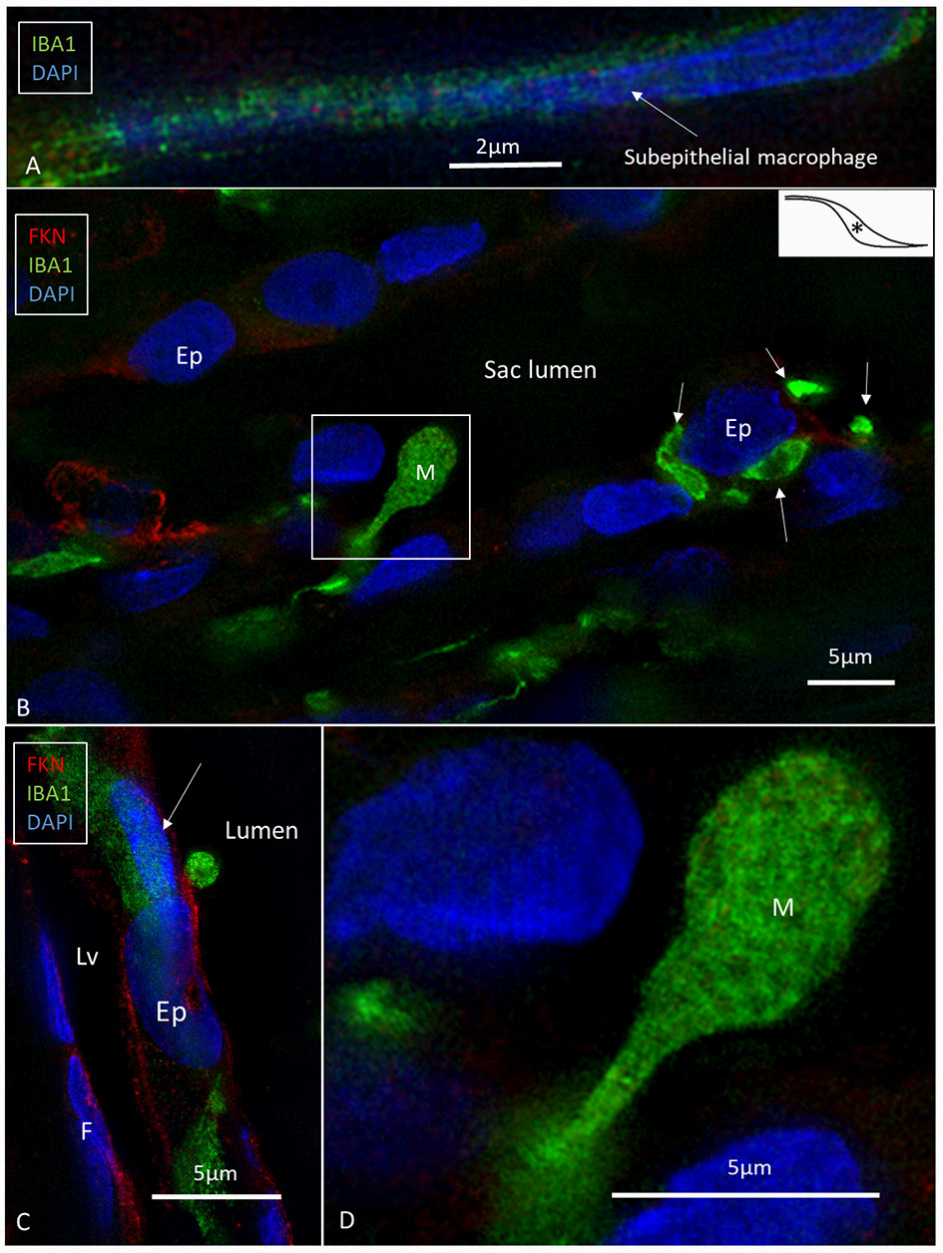
SR-SIM of human ES near the external aperture of the VA. **(A)** An elongated sub-epithelial cell expressing IBA1. **(B)** IBA1-positive cells are located both sub-epithelial and within the epithelium (Ep). One cell seems to have been loosened and expelled into the lumen of the ES. The framed area is magnified in **(D)**. One epithelial cell is surrounded by several IBA1-positive cells (arrows). **(C)** An IBA1 cell is located next to an epithelial cell (arrow). Sub-epithelial fibrocytes (F) and epithelium express fractalkine. Lv, lymphatics; D, The framed area in B magnified to show an IBA1 cell, presumably being expelled into the lumen; M, Macrophage. ^*^Shows location in the ES.

**Figure 4:**
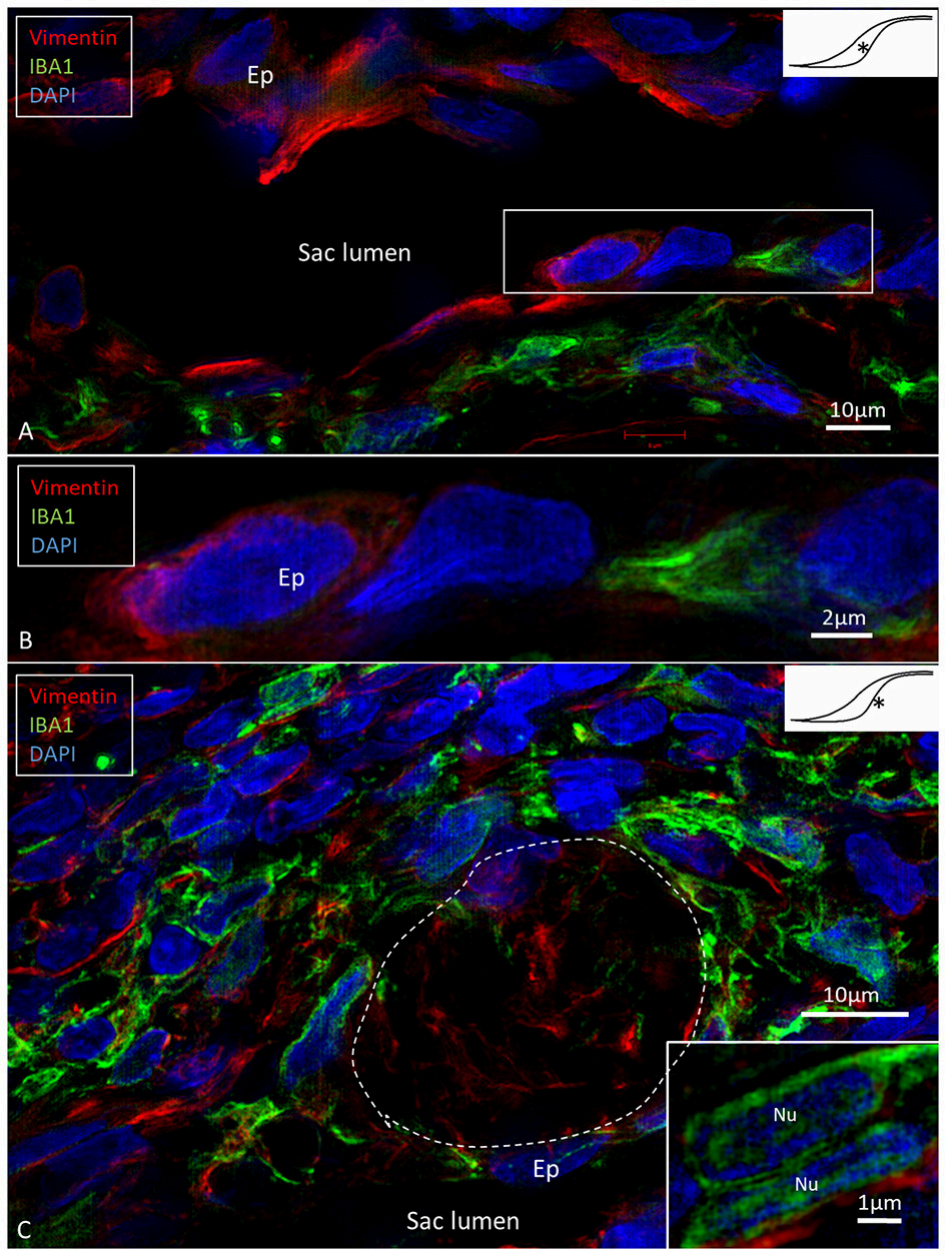
SR-SIM of epithelial conglomerate at the intra-osseous part of the ES. **(A,B)** Some epithelial cells (Ep) express the intermediate filament vimentin. **(C)** The accumulation of IBA1 cells is visible beneath the epithelium and around several vimentin-positive cells. Inset, The IBA1 cells show characteristic nuclear (Nu) staining. ^*^shows location in the ES.

**Figure 5:**
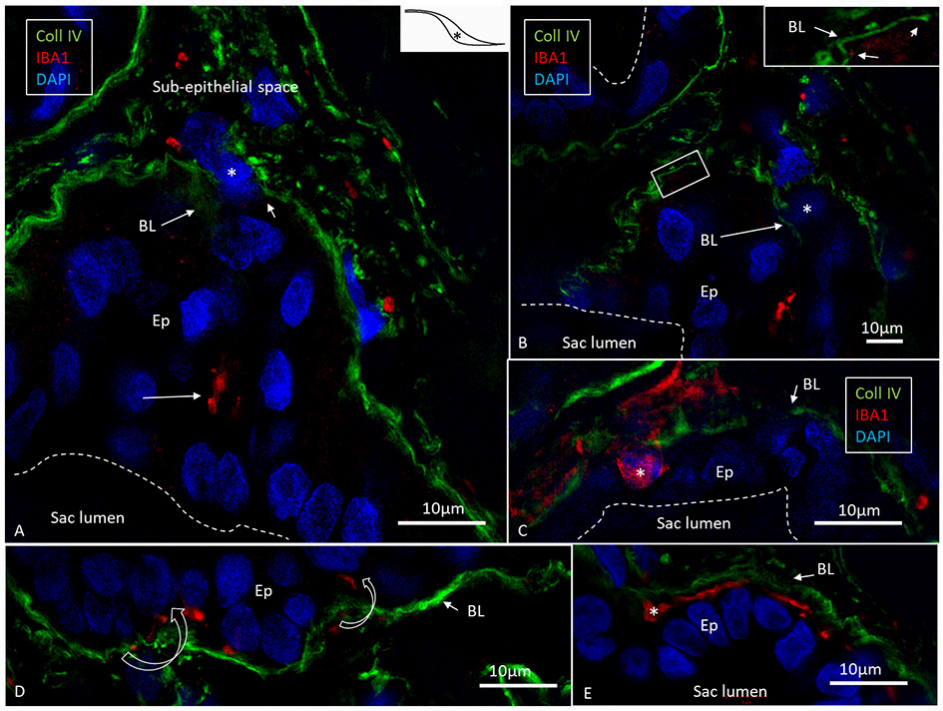
Expression of IBA1 cells and collagen IV. SR-SIM of the intra-osseous part of the ES showing several epithelial tubules. **(A)** IBA1 cells are located in the epithelium (Ep) as well as in the sub-epithelial space. **(B)** Adjacent section shows discontinuous basal lamina (BL) and trans-epithelial migration of one IBA1-neagtive cell (^*^). **(C)** Large IBA1 cell (^*^) crosses the BL and epithelium. **(D,E)** IBA1 cells located between the BL and epithelial cells. Note the extensive fragmentation of BL collagen IV expression in the sub-epithelial space. ^*^Shows location in the ES.

**Figure 6:**
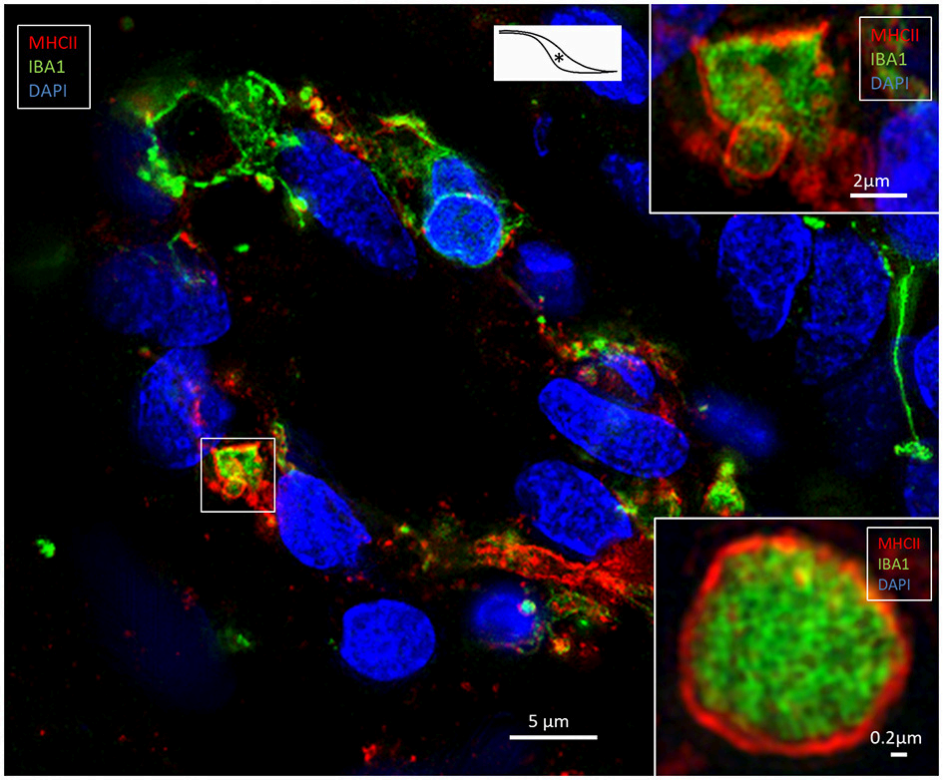
SR-SIM of an epithelial tubule in the intra-osseous part of the ES. Several epithelial cells express IBA1; some also express MHCII. The framed area is magnified in the top inset. The lower inset shows a similar cell, but it is located in the sub-epithelial space. ^*^Shows location in the ES.

**Figure 7:**
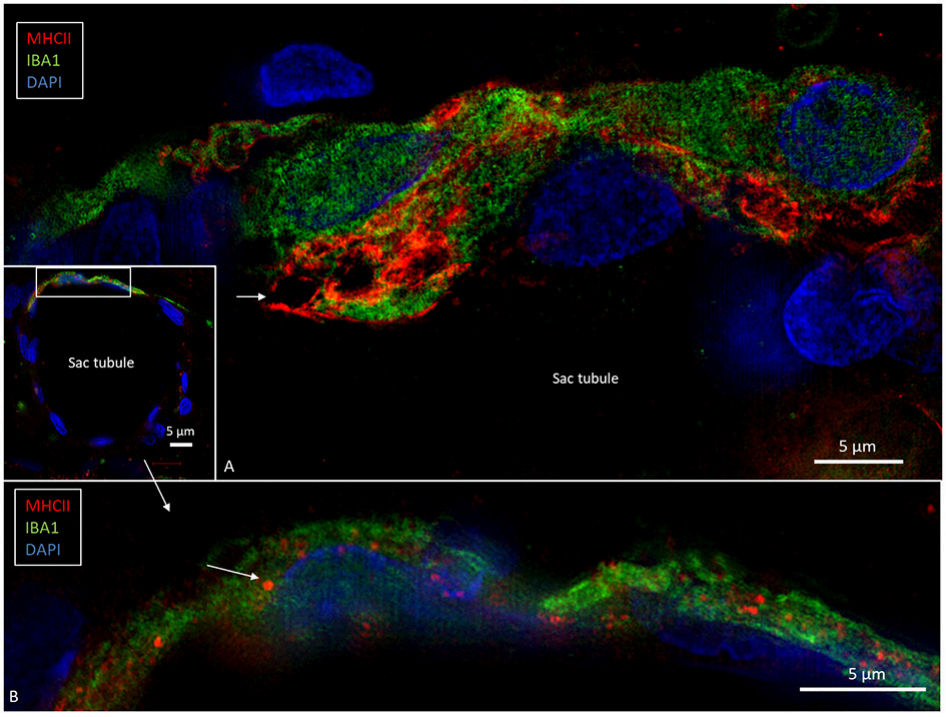
SR-SIM of epithelial tubules in the intra-osseous part of the ES. **(A)** Several IBA1-positive cells are present in the epithelium and seem to constitute an integral part of the epithelium. The cells express MHCII. Large endocytic vesicles (arrow) express MHCII. **(B)** The framed area of the inset is shown under a higher magnification; some epithelial cells express IBA1 as well as MHCII (arrow).

**Figure 8:**
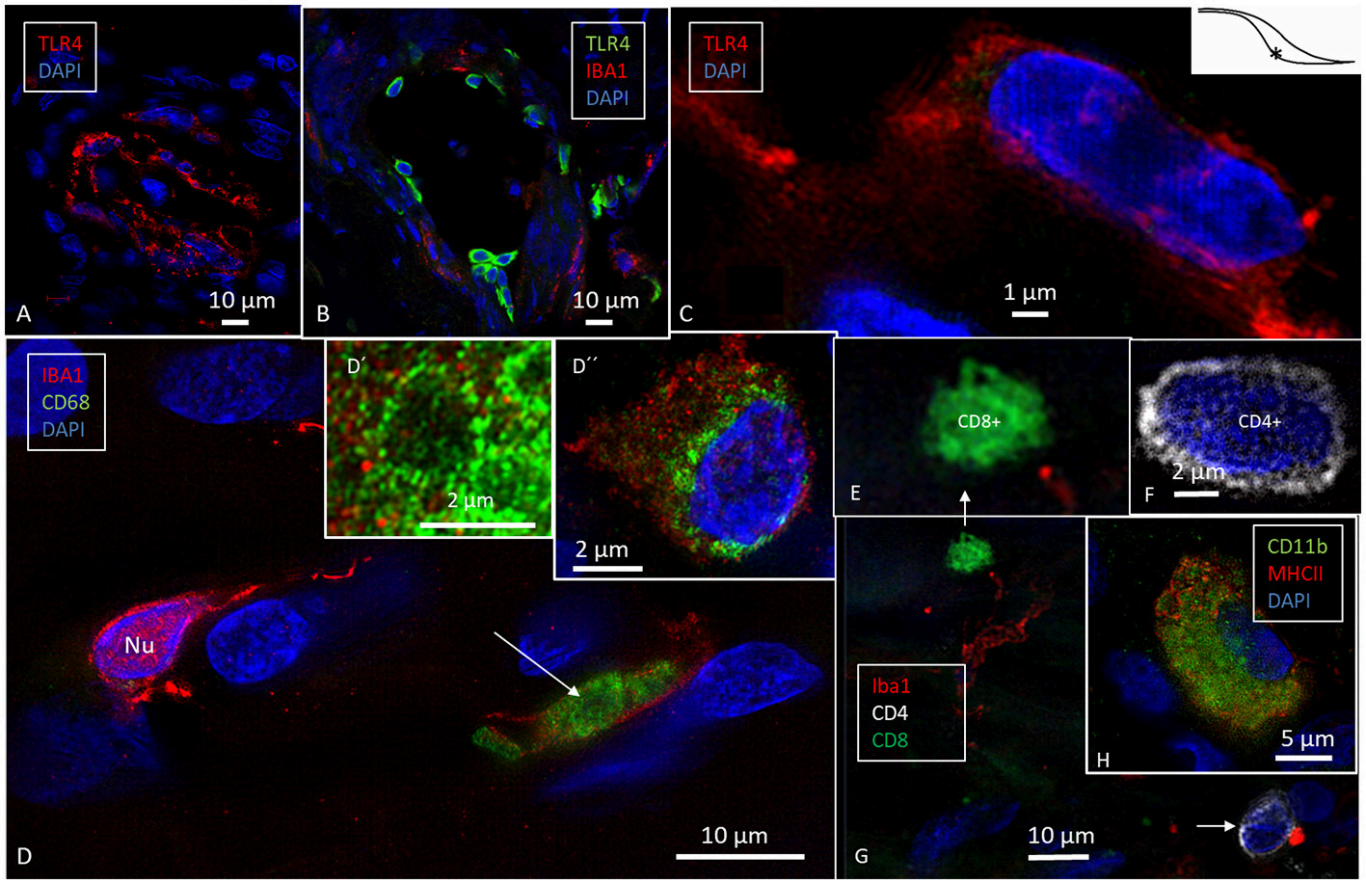
SR-SIM of cells expressing the toll-like receptor 4 (TLR4, **A–C**), CD68 **(D)**, and CD11b **(H)** in the human ES. TLR4 is expressed both in the cell membrane and in the cytoplasm **(C)**. **(D)** CD68 and IBA1 co-expression in the sub-epithelial cells (arrow, inset). Inset **D′** shows CD68+ cell in a higher magnification and **D″** shows an IBA1 cell with perinuclear distribution of CD68. **(E–G)** show sub-epithelial cells expressing lymphocyte markers CD8+ and CD4+ associated with IBA1 cell (arrow, four channels used). **(H)** One cell co-expresses CD11b and MHCII. ^*^Shows location in the ES.

SR-SIM and confocal microscopy showed a large number of IBA1-expressing cells in all the investigated parts of the ES (Figures 2–8). IBA1 cells resided both in the sub-epithelial tissue and in the epithelium. A surprisingly large number of perisaccular connective tissue cells expressed IBA1 (Figure 2C). These cells were elongated and branched, while others were stellate-like with long cross-sectioned branches. The cells' nuclei expressed IBA1, which made them distinguishable from fibrocytes (Figures 2A,B, 3A). In the epithelium, the cells were located between and beneath the epithelial cells; in addition, the epithelial cells themselves expressed IBA1 (Figures 3C, 6, 7). Some IBA1 cells showed signs of trans-epithelial migration, and were expelled into the sac lumen (Figures 3B,D). Large aggregations of IBA1 cells could be demonstrated at some places (Figure 4). The intra-epithelial location of the IBA1 cells could be documented by collagen IV immunostaining of the basal lamina (BL; Figure 5). The BL was often irregular and fragmented at these sites, with accumulated remains (Figure 5).

The epithelial cells expressed the chemokine fractalkine (Figure 2B). The expression varied between different cells and was mostly diffuse and intra-cytoplasmic. Some sub-epithelial fibrocytes also expressed fractalkine (Figure 3C).

### Expression of IBA1 and Major Histocompatibility Complex Type II

Many epithelial (Figures 6, 7) and non-epithelial cells (Figure 6, Supplementary Figure 1) co-expressed IBA1 and MHCII. Those appearing in the epithelium appeared to be both free intra-epithelial cells and true epithelial cells, especially in the secretory-like tubules of the intermediate ES (Figure 6). Among the epithelial cells, MHCII was mostly expressed in the apical membrane. Some of these cells contained large vesicles (2–3 microns) that occasionally opened to the endolymph space (Figure 7). The epithelial IBA1 cells also contained small aggregates of MHCII scattered in the cytoplasm (Figure 7B).

### Expression of Toll-Like Receptor 4, CD68, and CD11b

High-resolution fluorescence microscopy also disclosed expression of toll-like receptor 4 (TLR4) among the sub-epithelial cells in the intermediate ES (Figures 8A–C). These cells did not co-express IBA1. TLR4 was expressed both in the cell membrane and in the cytoplasm. In some cells the nuclei also expressed TLR4 (Figure 8C). A few sub-epithelial cells expressed CD68 (Figure 8D), which was occasionally co-expressed with IBA1. Several migrating cells expressed CD68 and CD11b together with MHCII (Figure 8H). Round cells expressing CD4 and CD8 were found in the ES, with more CD4+ than CD8+ cells (Figures 8E–G). Physical interaction between a CD4+ and an IBA1 cell was noticed (Figure 8G).

### IBA1 and Major Histocompatibility Complex Type II in the Human Cochlea

**Figure 9:**
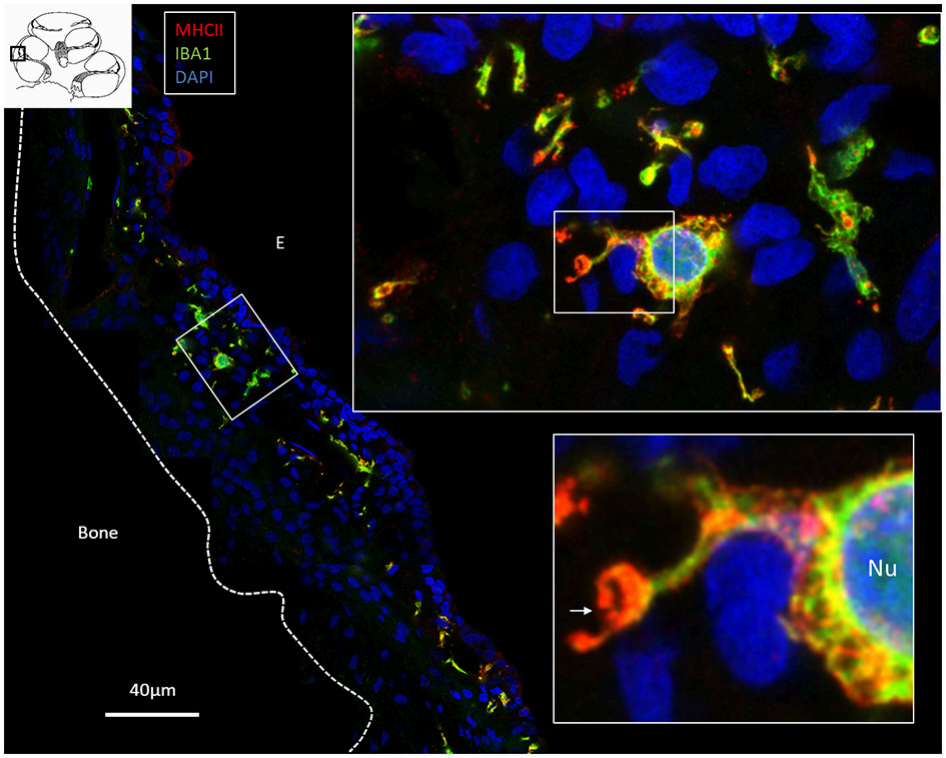
Confocal microscopy and SIM of the human stria vascularis. Cells co-express IBA1 and MHCII. The framed area is magnified in the insets. Several IBA1 cells are located in the stria epithelium, but few are found in the spiral ligament. Insets, The nuclei (Nu) express IBA1. The cell membrane (arrow) and the cytoplasm contain vesicles expressing MHCII. E, Endolymph.

To realize if IBA1 cells in the human cochlea also expressed MHCII, archival sections were stained. Numerous IBA1-positive macrophages expressed MHCII in the stria vascularis and spiral ganglion (Figure 9). The cells contained cytoplasmic aggregates of MHCII (Figure 9, lower inset) and their slender processes often embraced the vessels. Fewer, but similarly stained cells were detected in the spiral ligament. TLR4 was expressed in the stria vascularis (not shown).

## Discussion

### Structured Illumination Microscopy and the Human Endolymphatic Sac

This is the first study to use super-resolution immunohistochemistry of the human ES, which showed the spectacular organization of resident IBA1-positive macrophages and the molecular expression in great detail. The large quantity of macrophages was unexpected, since most of the sub-epithelial cells seemed to represent fibrocytes, while the SR-SIM immunohistochemistry showed them to be resident macrophages. The IBA1 marker protein (ionized calcium binding adaptor molecule 1) is an actin cross-linking protein associated with membrane ruffling and phagocytosis (35, 36). It is expressed in both reactive and quiescent microglial cells (37). Previous studies have yielded similar findings in the human inner ear; several resident macrophages were found to be positive for markers CD163, IBA1, and CD68 (1, 2). The cells were localized in the inner ear under steady-state conditions (19, 38–40), suggesting that the inner ear contains immune-competent cells that may activate both innate and adaptive immune responses. IBA1 was typically expressed in the nuclei, the cytoplasm, and in small cytoplasmic specializations against adjacent tissue (2, 41). The immune state of the inner ear was not believed to be influenced by the benign cranial tumor in the internal ear canal. Cochlear macrophages are thought to derive from bone marrow myeloid precursors, and they do not seem to undergo self-renewal during life (42). The term “microglia” may therefore not be an appropriate term since these cells have different ontogeny, morphology, and surface markers (43).

### Vestibular Schwannoma and Tissue Collection

The collected tissue was from patients with benign tumors located in the internal acoustic canal. Therefore, one cannot rule out the possibility that the immune state of the inner ear could be influenced by this pathology, even though located at some distance. However, the similar findings of IBA1 cells made by O'Malley et al. (1) using alternate histological techniques would seem to refute this. Their material was from archives of different donated temporal bones with no tumors. Likewise, some of these findings were made in experimental animals (Okano et al.) (20).

### Blood-Labyrinth Barrier and Fractalkine Signaling (CX3CL1/CX3CR1)

The human inner ear is sheltered by hard bone, and it is separated by a so-called blood-labyrinth barrier, which is equivalent to the blood-brain barrier and may be crucial for the immune defense. In the brain, bone marrow-derived macrophages are restricted to regions lacking a blood-brain barrier. The barrier consists of tight junctions separating the endolymph from the extracellular tissue. The spiral ganglion and the ES contain fenestrated vessels suggesting that they are not embraced by the blood-labyrinth barrier (44). Consequently, blood-borne leukocytes may migrate more readily into the ES. Hirose et al. and Kaur et al. (18, 40) discovered that cochlear injury and selective loss of cochlear hair cells increase the number of marrow-derived monocyte/macrophage lineage cells in the cochlea, including the auditory nerve, and spiral ligament. Fractalkine, a chemokine normally found in neural cells, was found to play a significant role for the attraction through the macrophages' receptor expression. Signaling induces chemotactic cell adhesion. As well it was essential to recruit macrophages by noise, ototoxic drugs, age, and diphtheria toxin induced selective hair cell degeneration (18, 39, 40, 43, 45–52). Mice lacking the receptor (CX3CR1) showed a reduced capacity to recruit macrophages (18). The chemokine seems to be expressed in both mice and human cochleae (2, 18). The present results indicate that fractalkine plays a similar role in the ES to attract macrophages to the epithelium for immune reaction. The verification of chemokine receptor expression in the ES macrophages has not been performed so far.

### Expression of MHC Class II in the Endolymphatic Sac and Cochlea

SIM revealed a strong expression of MHCII in IBA1 cells in the ES. Surprisingly, the macrophages populating the stria vascularis (and spiral ligament) and spiral ganglion also expressed MHCII (Figure 9). These molecules are essential to initiate specific immune responses by presenting antigens to CD4+ T helper cells and interaction with antigen-specific B cells. Earlier studies showed no MHCII expressing cells in the inner ear, unless induced by experimental inflammation or γ-interferon (53, 54). It seemed consistent with the view that the inner ear lacks immune responsiveness. Okano et al. (20) however, found bone marrow-derived cells, both in the vestibular end organs and the in ES expressing MHCII molecules. Altermatt et al. (55) found a few lymphoid cells expressing MHCII in the epithelial layer in the ES collected post-mortem. Here, MHCII was strongly expressed on the apical cell membrane and vesicles in several tubular epithelial cells. This was remarkable, but it is known that intestinal epithelial cells may express MHCII to play roles in mucosal immunology, modulation, and disease (56, 57). These molecules together with antigen cargo can be expressed on the cell surface in thymus epithelial cells (58). In this journal Wosen et al. (59) reviewed existing data on the interaction and regulation of epithelial MHCII molecules in health and disease. Our findings of the co-expression of IBA1 and MHCII in epithelial cells and the trans-epithelial migration propose that there is an uptake and processing of antigens from the ES lumen. Signs of intra-epithelial immune cell interaction were earlier demonstrated by transmission electron microscopy (5).

### TLR4, CD4, CD68, and CD11b/Integrin Alpha M

Möller et al. (17) studied the gene expression of the humoral innate immune-system in the human ES and found genes equivalent to that of the mucosa-associated lymphoid tissue (MALT). The expression was verified by immunohistochemistry, suggesting antigen recognition, and processing for the initiation of immune responses. Multiple key elements of both the cellular and humoral innate immune-system were expressed, including toll-like receptors (TLRs) 4 and 7, beta-defensin, and lactoferrin. According to Möller et al. (17), the ES may provide defense not only against bacteria and viruses but also against fungal infection of the inner ear. Altermatt et al. (55) found immunostaining with antibodies against the CD4 and CD8 antigens and revealed a predominance of CD4+ T lymphocytes in the ES. According to Yamada et al. (60), the TLRs 2, 3, 4, and 9 are highly expressed in human ES fibroblasts and can produce cytokines and chemokines in response to the TLR ligands, thereby playing a role during the initiation of immune responses. The findings suggest that the human ES epithelium may trap and engulf intraluminal material reinforced by the TLR 4 signaling. SR-SIM also detected both CD4+ and CD8+ cells that also interacted with macrophages. Danckwardt-Lillieström et al. (61) showed ultrastructural evidence of cytotoxic lymphocyte activity in the ES epithelium of a patient with Meniere's disease. It was associated with epithelial cell adhesion and degeneration suggesting that toxic lymphocytes may be directed against own ES cells with on-going autoimmune activity in some cases with this disease.

CD68 was localized in some IBA1-positive cells. CD68 is a 110 kD transmembrane glycoprotein that is highly expressed in human monocytes and tissue macrophages (62). It may promote phagocytosis and recruit macrophages (63, 64) and it is widely used as a macrophage marker. CD68 can bind to organ-specific lectins to accomplish these cells homing to particular sites. Macrophages can upregulate CD68 in response to inflammation (65) and can bind to apoptotic cells, possibly exerting antigen processing/presentation. The exact function of CD68 in the inflammatory responses remains somewhat elusive (66). A recent study reported few CD68-positive cells in the ES, mainly in the lumen, while the IBA1-positive cells were more consistent in the ES epithelia and connective tissue (1). The present study seems to have confirmed these findings; CD11b/Integrin alpha M was also expressed in MHCII expressing cells in the ES and spiral ganglion (not shown here). CD11b, also known as macrophage-1 antigen or complement receptor 3, may bind to molecules on the surface of invading bacteria leading to phagocytosis.

**Figure 10:**
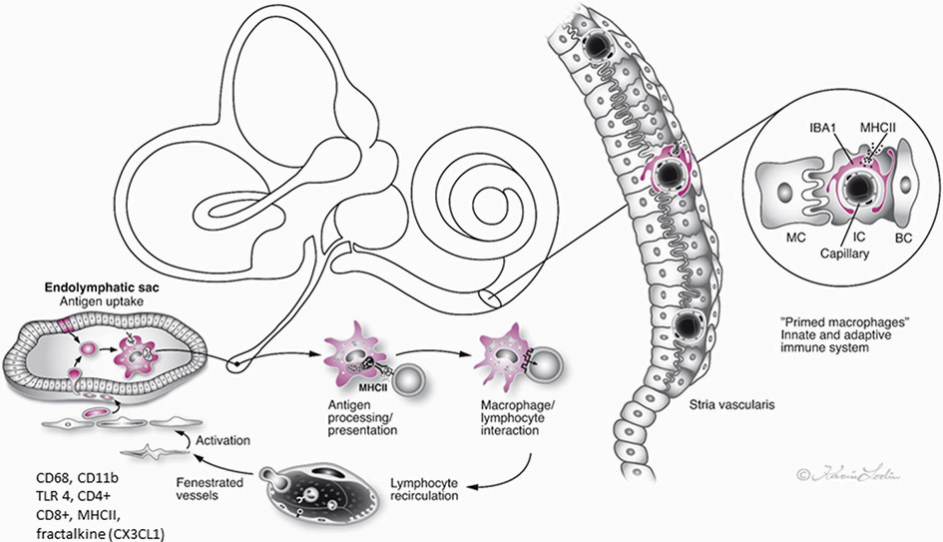
Hypothetical representation of the immune interaction between the endolymphatic sac (ES) and the inner ear in man. The ES receives antigens and waste material via the endolymphatic duct, which activates resident macrophages/monocytes that then migrate into the sac lumen. After phagocytosis, cells migrate back into the perisaccular tissue for antigen presentation and immune processing. MHC class II molecules, reinforced by TLR4, and under IFNγ stimuli, bind to endocytosed, and degraded peptides within the cytoplasm and transport the peptides to the cell membrane for presentation to CD4+ T cells. The lymphocytes recirculate into the bloodstream to “prime” the inner ear cells. The immune activation of the inner ear is located “off-site” to avoid pro-inflammatory activation near the vulnerable hair cells. The chemokine fractalkine assists in attracting macrophages to the ES (2, 4, 5, 10, 17).

The present investigation shows the large population of macrophages in the human ES. Their capacity for immune processing and their possible links to other cochlear and vestibular macrophages need further elucidation. We speculate that the immune cell machinery in the ES protects the sensory organs, thereby avoiding a full-scale release of pro-inflammatory mediators and antimicrobial activity within the bony enclave of the inner ear (Figure 10). Clearly, more studies are needed to fully understand the function of this hidden and intriguing part of the human inner ear as well as its role in inner ear disease.

## Conclusions

High-resolution structured illumination microscopy (SR-SIM) was used to analyze the molecular expression of potential immune activity in the ES in the human inner ear. From these results, we conjecture that human hearing and balance organs may be immunologically protected by the ES. Thus, the potentially harmful induction of inflammatory activity near the vulnerable sensory structures may be circumvented.

## Author Contributions

Processing of tissue, immune staining and SR-SIM were performed by WL and CN. Most of the writing was performed by HR-A and CN. ND-L collected the ES specimens. GL together with HR-A, WL, and CN planned the study.

### Conflict of Interest Statement

The authors declare that the research was conducted in the absence of any commercial or financial relationships that could be construed as a potential conflict of interest.

## Supplementary Material

Supplementary Figure 1 SR-SIM of cell interaction in the sub-epithelial space of the human ES near the external aperture of the vestibular aqueduct (VA). **(A)** An elongated IBA1 cell physically interacting with a cell expressing MHCII. The framed area is shown under higher magnification in inset **A'**. Both cells' nuclei (Nu) show different protein concentrations. The cell membranes express MHCII. A similar interaction is visible in **A”**. **(B)** A sub-epithelial macrophage shows intracytoplasmic expression of MHCII. The framed area is magnified in E and shows globular protein expression. **(C)** A long sheet of interacting flat cells co-express IBA1 and MHCII near the luminal region of the ES. **(D)** A sub-epithelial macrophage shows membrane expression of MHCII. **(E)** Higher magnification of framed area in **B**.

## Abbreviations

ED: endolymphatic duct
ES: endolymphatic sac
E: endolymph
EDTA: ethylene-diamine-tetra-acetic acid
SR-SIM: super-resolution structured illumination fluorescence microscopy
IBA1: ionized calcium binding adaptor molecule 1
VA: vestibular aqueduct
MHCII: major histocompatibility complex type II.
