# Supplementary figures and images for: The Human Endolymphatic Sac and Inner Ear Immunity: Macrophage Interaction and Molecular Expression
Source: Front Immunol. 2019 Feb 1;9:3181. doi: 10.3389/fimmu.2018.03181 (PMC6367985; doi:10.3389/fimmu.2018.03181)

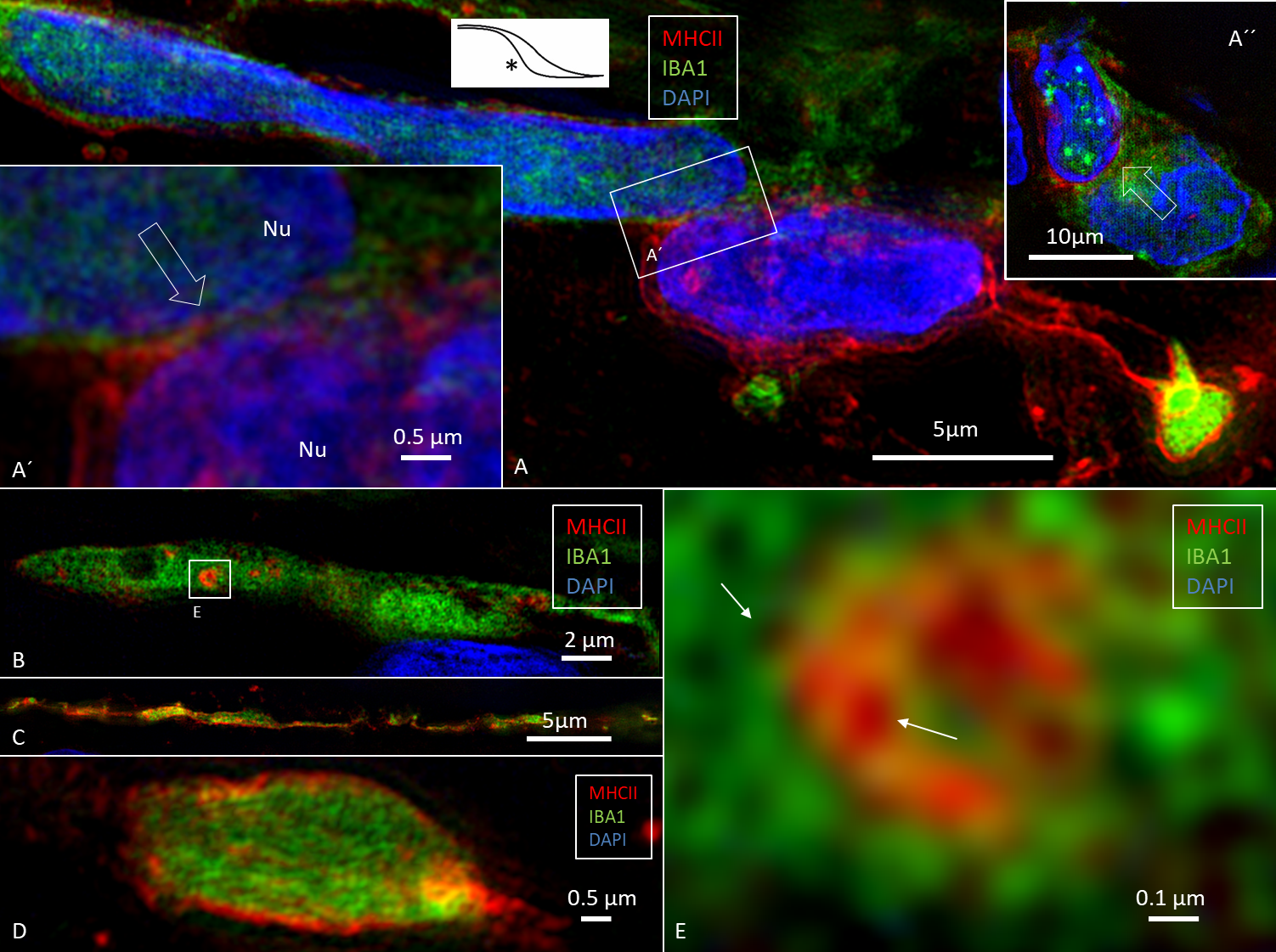

Supplement: Supplementary Figure 1 — SR-SIM of cell interaction in the sub-epithelial space of the human ES near the external aperture of the vestibular aqueduct (VA). (A) An elongated IBA1 cell physically interacting with a cell expressing MHCII. The framed area is shown under higher magnification in inset A'. Both cells' nuclei (Nu) show different protein concentrations. The cell membranes express MHCII. A similar interaction is visible in A”. (B) A sub-epithelial macrophage shows intracytoplasmic expression of MHCII. The framed area is magnified in E and shows globular protein expression. (C) A long sheet of interacting flat cells co-express IBA1 and MHCII near the luminal region of the ES. (D) A sub-epithelial macrophage shows membrane expression of MHCII. (E) Higher magnification of framed area in B. [file Image_1.tiff]
